# Electrospun Metal Oxide Nanofibers and Their Conductometric Gas Sensor Application. Part 1: Nanofibers and Features of Their Forming

**DOI:** 10.3390/nano11061544

**Published:** 2021-06-11

**Authors:** Ghenadii Korotcenkov

**Affiliations:** Department of Theoretical Physics, Moldova State University, 2009 Chisinau, Moldova; ghkoro@yahoo.com

**Keywords:** electrospinning, principles, hollow nanofibers, core-shell structures, modification, blending, doping, advantages, limitations

## Abstract

Electrospun metal oxide nanofibers, due to their unique structural and electrical properties, are now being considered as materials with great potential for gas sensor applications. This critical review attempts to assess the feasibility of these perspectives. The article in Part 1 discusses the basic principles of electrospinning and the features of the formation of metal oxide nanofibers using this method. Approaches to optimization of nanofibers’ parameters important for gas sensor application are also considered.

## 1. Introduction

**Figure 1:**
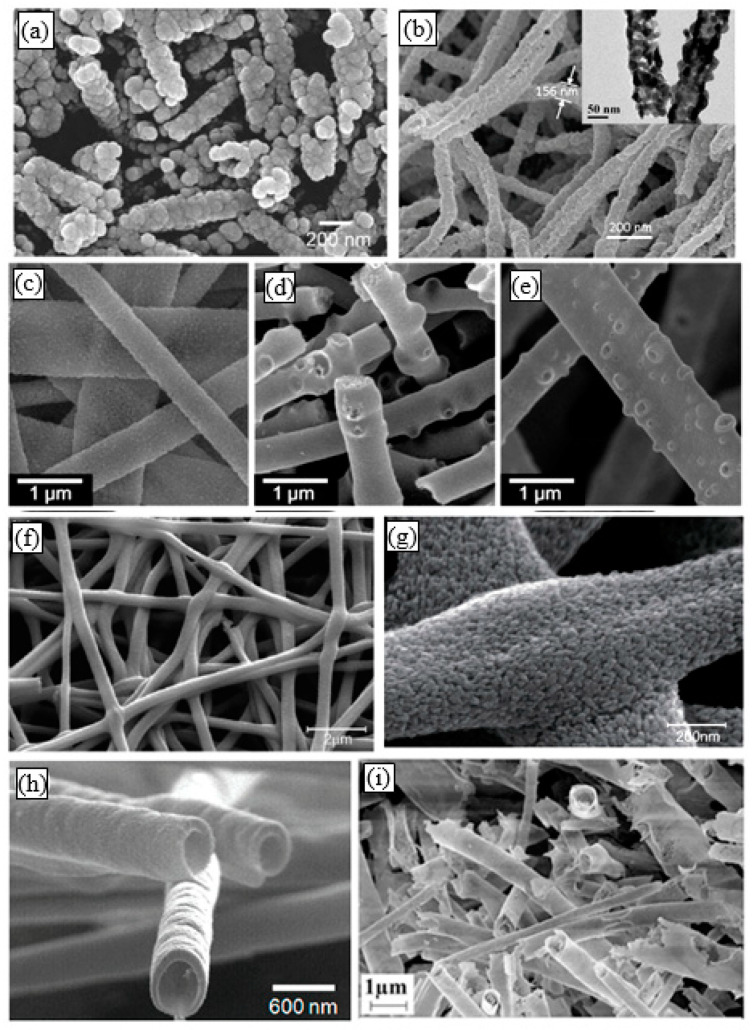
Typical SEM images of electrospun metal oxide nanofibers (NFs): (**a**) SnO_2_ NFs, (**b**) In_2_O_3_ NFs; (**c**–**e**) WO_3_ NFs with different porosity (*T_cal_* = 200–500 °C); (**f**,**g**) TiO_2_ NFs; (**h**) Hollow ZnO NFs (*T_cal_* = 500 °C); (**i**) Hollow Fe_2_O_3_ NFs (*T_cal_* = 650 °C). (**a**) Reprinted with permission from [25]. Copyright 2010 Elsevier; (**f**,**g**) Reprinted with permission from [26]. Copyright 2013 RSC; (**h**) Reprinted with permission from [27]. Copyright 2009 ACS; (**i**) Reprinted with the permission from [28]. Copyright 2019 Elsevier; (**b**) Reprinted from [29]; (**c**–**e**) Reprinted from [30].

In recent years, amid increased attention to metal oxide one-dimensional (1D) nanomaterials such as nanowires or nanotubes [1,2,3,4,5,6,7], noticeable interest is shown in metal oxide nanofibers (NFs). Although in nature they differ from classical 1D nanomaterials, in the scientific literature, they often refer to 1D nanomaterials [8,9,10,11]. Unlike classical 1D nanomaterials, which are monocrystalline in nature, metal oxide nanofibers are amorphous or polycrystalline. A typical view of metal oxide nanofibers is shown in Figure 1.

Earlier in [1,2,3,4,5,6,7], it was shown that one of the most promising fields of application of metal oxide 1D nanomaterials is the development of conductometric gas sensors based on them. As will be shown below, metal oxide nanofibers are also promising materials for these applications since gas-sensitive layers based on them are characterized by high porosity and large surface-to-volume ratio, which is typical for materials developed for highly sensitive and high-speed devices [9,12]. This means that the use of nanofibers can provide improved sensor performance. It is important to note that a porous structure made out of nanofibers is a system where the pore size and shape can change easily, in contrast to conventional rigid porous structures, made using conventional thin- and thick-film technologies. Therefore, the membranes assembled by nanofibers have structural properties that are excellent for gas sensor applications. In addition, compared to real one-dimensional metal oxide nanostructures such as nanorods, nanowires, and nanotubes, nanofibers are continuous with high flexibility prior to calcination. Many additional functions can also be incorporated into nanofibers to expand their applications [13,14,15,16,17]. These features of nanofibers open up additional possibilities for creating a gas-sensitive matrix with optimal properties.

By now, a huge amount of information has been accumulated concerning the development of gas sensors based on metal oxide nanofibers. Moreover, quite a few reviews have already been published devoted to the consideration of electrospinning technology [15,16,17,18,19,20] and its use in the development of gas sensors [9,10,13,14,15,21,22,23,24]. However, these reviews did not aim to assess the real advantages and disadvantages of these materials, which are manifested in the development of gas sensors based on them, as was done in [7] in relation to 1D and 2D nanomaterials. In this review, we will try to fill this gap.

Taking into account a wide variety of metal oxides prepared in the form of nanofibers and the large amount of information obtained during the development of gas sensors based on them, our review, “Electrospun metal oxide nanofibers and its conductometric gas sensor application”, is divided into two parts: Part 1: Nanofibers and features of their forming, and Part 2: Gas sensors and their advantages and limitations. The first part, i.e., this article first briefly discusses the basic principles of electrospinning used for the formation of polymer fibers. Further, the results related to the formation of metal oxide nanofibers are considered in detail. This consideration was carried out in terms of the formation of nanofiber-based gas-sensing materials. In the following Part 2 of this article, approaches to the fabrication of gas sensors are considered, as well as the results of analysis of the characteristics of fiber-based gas sensors. It then provides a detailed analysis of the drawbacks that may limit the use of electrospinning technology in the development of gas sensors. Some approaches to solving these problems are also suggested in Part 2. Finally, the summary provides an insight into the future prospects of electrospinning applications for the development of gas sensors.

## 2. General Aspects of Electrospinning

Nanofibers (NFs) can be formed using a variety of approaches [17,31,32,33]. However, the most common and most promising method at the moment is electrospinning [14,15,16,17,18,19,20,33]. Electrospinning is considered by many to be the best method for large-scale preparation of NFs compared to other available methods due to its features such as ease of handling, minimal solution consumption, high flexibility for producing long and continuous NFs, controlled NF diameter, low cost, and ease of processing equipment [19,20,34].

**Figure 2:**
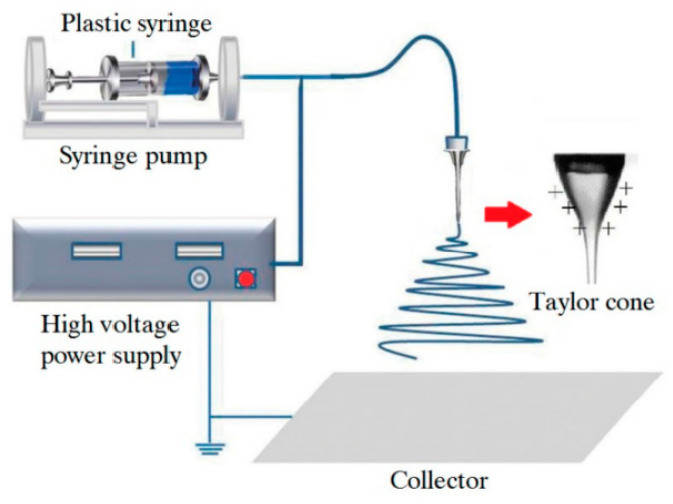
Schematic diagram for the demonstration of polymer nanofibers produced by electrospinning. Reprinted from [36].

Initially, electrospinning was developed for polymers [35]. A typical setup for electrospinning consists of a high-voltage power supply (10–70 kV), a spinneret with a metallic needle, a solution reservoir, and a grounded collection device, as shown in Figure 2. A solution of polymer, polymer melt, or composite is loaded into the syringe, and this viscous liquid is driven to the needle tip by a syringe pump, forming a droplet at the tip.

Although a spinneret with a metallic needle is convenient for applying a charge to the solution, the process also works if a high voltage is applied to the solution using a special electrode with a nonconducting spinneret and needle. When the repulsive force, induced by the charge distribution on the drop surface, is balanced with the surface tension of the liquid, the liquid drop distorts into a conical shape. Once the repulsive force exceeds the surface tension, a charged jet of liquid is ejected from the tip of the cone and moves toward a grounded electrode. Unlike conventional spinning, the jet is only stable near the tip of the spinneret, after which the jet is subject to bending instability. Whether the jet will form a continuous fiber or disperse into droplets depends on polymer molecular weight, polymer chain entanglement, and the solvent used in the process (specifically, its evaporation rate). The liquid jet will stay in a single stream status if molecular cohesion overcomes electrostatic repulsion or break up otherwise. If the resultant is collected as droplets, then the process should be called electrospraying instead of electrospinning, where the liquid jet breaks up into multiple streams. The theory for electrospinning one can find in [37,38,39,40].

Generally, the electrospun fibers are being deposited on a fixed collector having a horizontal or vertical arrangement (see Figure 2). Usually, the nanofiber is randomly deposited in a spiral manner on the surface of the collection device. The collector, as a rule, is a metal foil, but it can be of any material and in any configuration according to the required final product. This setup can be boxed so that the atmospheric humidity can be controlled and changed as required. With the expansion of this technology, several research groups have developed more sophisticated systems [17,20,33,41,42,43,44].

### 2.1. Conditions Required for the Successful Implementation of Electrospinning

This section may be divided into subheadings. It should provide a concise and precise description of the experimental results, their interpretation, as well as the experimental conclusions that can be drawn.

**Figure 3:**
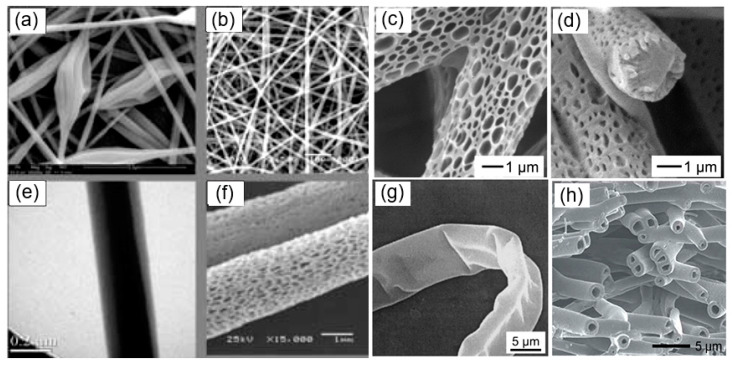
Different polymer fiber morphologies: (**a**) beaded; (**b**) smooth; (**c**,**d**,**f**) porous, (**e**) core-shell; (**g**) ribbon-like, and (**h**) hollow fibers. (**a**,**b**,**e**,**f**) Reprinted from [20]; (**c**,**d**) Reprinted with permission from [49]. Copyright 2018 American Chemical Society; (**g**) Reprinted with permission from [50]. Copyright 2001 Wiley-VCH; (**h**) Reprinted with permission from [51]. Copyright 2014 Royal Society of Chemistry.

The experiment showed that for the successful implementation of electrospinning, it is necessary to fulfill several conditions [17,45,46]:

- Although electrospinning could use molten polymers [47], researchers generally choose polymer solutions for electrospinning. Therefore, the solvent used must be capable of dissolving the polymer material intended for nanofibers preparation. For example, common polymers, such as polyethylene (PE), polypropylene (PP), and polyamide (PA), can only be dissolved in certain solvents at high temperatures. However, it must be borne in mind that the solvent with a high solubility parameter does not necessarily produce a solution suitable for electrospinning. When choosing solvents, it should also be borne in mind that most polymer solvents that can be used for these purposes are harmful to the health of workers. Some polymer solvents combinations commonly used to produce nanofibers by electrospinning are listed in Table 1;
- The vapor pressure of the solvent should be suitable so that it evaporates quickly enough for the fiber to maintain its integrity when it reaches the target but not too quickly to allow the fiber to harden before it reaches the nanometer range. However, very high volatility is not suitable for fiber spinning as the jet can solidify immediately after exiting from the spinneret. If the volatility is too low, the fibers will still be wet when they are deposited on the collector;
- The viscosity and surface tension of the solvent should be carefully balanced. They must neither be too large nor be too small. It was established that smooth fibers are being produced when the product of intrinsic viscosity (η) and polymer concentration (c), known as Berry’s number, Be = η × c, is greater than a certain critical value, which is characteristic of the polymer used. In the case of low-viscosity and low concentration liquids instead of nanofibers, small droplets are formed as a result of the varicose breakup of the jet. One should note that high solution concentration and large viscosity and surface tension of solvents for the electrospinning process are also not optimal. In this case, the formation of continuous fibers is prohibited due to the impossibility of maintaining the flow of solution at the tip of the needle. In optimal conditions, a solid fiber is generated instead of breaking up into individual drops due to the electrostatic repulsions. As the charged jet accelerates toward regions of lower potential, the solvent evaporates while the entanglements of the polymer chains prevent the jet from breaking up. This results in fiber formation. Typical SEM images of polymer fibers are shown in Figure 3. The fiber diameter can vary from a few nanometers to a few micrometers. Within a suitable range, decreasing polymer concentration tends to produce finer fibers;
- The power supply must offer a voltage large enough to overcome the viscosity and surface tension of the polymer solution to form and sustain the jet from the pipette;
- Regardless of the setup configuration used, the collector must be located at a certain distance from the needle. The gap between the pipette and grounded surface should not be too small to create sparks between the electrodes but should be large enough for the solvent to evaporate in time to form fibers. Typically, this distance can be varied from 9 to 25 cm.

### 2.2. Advantages and Limitations of Electrospinning Technology

**Figure 4:**
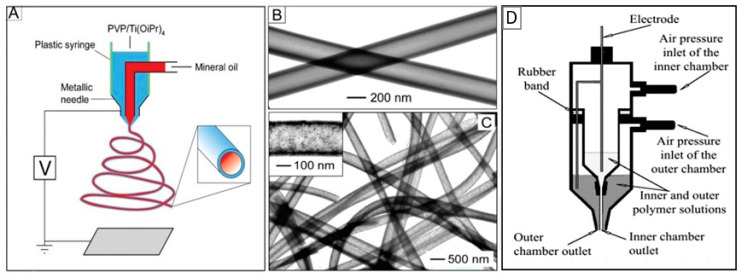
(**A**) Schematic illustration of the setup for coaxial electrospinning; (**B**, **C**) SEM images of TiO_2_ hollow nanofibers; (**D**) experimental setup used for coaxial electrospinning of core/sheath nanofibers. In this installation with a non-conductive spinneret and needle, unlike the traditional approach, a high voltage is applied directly to the solution using a special electrode. (**A**–**C**) Reprinted with permission from [55]. Copyright 2004: Wiley; (**D**) Reprinted from [56]. Copyright 2003 Wiley.

As you can see, the device for electrospinning shown in Figure 2 has a single nozzle. This is the most common conventional electrospinning technique, which uses a single viscous polymer solution in combination with other required materials to produce nanofibers. This method is inexpensive and easy to use. However, there are several limiting factors for this technique. One of the key limitations is that it cannot be used with non-spinning solutions. To overcome this limit, a new technique called coaxial electrospinning [41,42,52] was developed (see Figure 4). Here, the two-layer nozzle consists of an outer capillary and a smaller inner capillary. This technique greatly expands the possibilities of electrospinning since it can be used to spin two different polymer-based solutions to make complex fibers [52,53,54]. It is important to note that the non-spinnable solution can also be used to form nanofibers by coaxial spinning.

A side-by-side method of electrospinning offers a different approach to using several polymer-based solutions in the nanofiber forming process [57]. Such devices have adjacent nozzles with two or more separated capillary chambers for extruding a mixture of different polymers with different properties [58]. This method reduces the complexity of coaxial spinning. In addition, multi-jet electrospinning is one of the best configurations for depositing multiple materials, covering large areas or depositing different layers on top of each other after appropriate drying steps [59].

**Figure 5:**
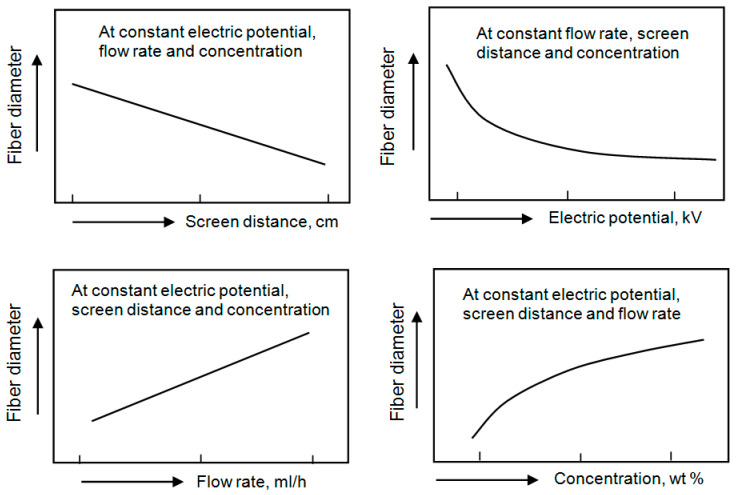
Influence of the process parameters on the fiber diameter obtained by the electrospinning method.

It is believed that the main advantage of the electrospinning technique is the ability to control the fiber diameter, high surface-to-volume ratio, and porosity of the formed nanofiber mat. However, as a result of extensive research on electrospinning, it was found that it is quite difficult to carry out such control. Nanofiber parameters such as fiber collectability, uniformity of fibers, average fiber diameter, fiber diameter distribution, and fiber porosity are highly dependent on a large number of processing parameters such as solution properties [60,61,62,63,64]. Processing parameters include molecular weight (M_w_), molecular weight distribution and architecture (branched, linear, etc.) of polymers, polymer concentration, solution viscosity, solution conductivity, and surface tension, flow rate, applied voltage, the working distance between the collector and the needle tip and ambient parameters such as temperature, air humidity and air velocity in the chamber [17,55,65,66,67]. The motion of the target screen (collector) also affects nanofiber parameters. The correlation between fiber diameter and electrospinning process parameters is shown in Figure 5 and discussed in Table 2. The data presented in Table 2 were extracted from [17,64,68,69,70,71].

From the above, it follows that for each electrospinning application, it is necessary to select suitable materials such as polymer and solvent and to optimize the spinning process in order to obtain nanofibers with a certain morphology and properties corresponding to the desired functions of nanofibers in the expected applications. At that, there are no secondary factors when optimizing the electrospinning process [17]. Even the conductivity of the solution and air humidity plays a significant role. For example, by increasing the conductivity of the solution, the carried charge can be increased, which can help in the stretching of polymer chains, promoting a decrease in the fiber’s diameter and avoiding the appearance of beads on the fibers. Relative humidity affects the rate of evaporation of the solvent and, therefore, the rate at which the jet solidifies [17]. The lower relative humidity results in the formation of finer fibers with a drier surface. However, if the relative humidity is too low, the solvent will quickly evaporate, preventing the extension of the jet. On the other hand, when the relative humidity reaches a high enough level, water vapor from the air can penetrate into the jet, causing morphological changes in the nanofibers. This process promotes pore formation in the fibers [68,69].

According to Lubasova and Martinova [72], the use of two solvent systems with different relative volatilities can also help achieve a porous structure in nanofibers during electrospinning. The temperature of the collector also has a strong effect on the physical structure of the porous electrospun nanofibers. Kim et al. [73] found that the influence of collector temperature on the structure and the density of the pores of the fiber depends on the boiling point of the solvent and the glass transition temperature (Tg) of the polymer. A more detailed explanation of the preparation and application of electrospun polymer fibers can be found in excellent review articles already published [17,36,45,70,71,74,75].

Other advantages of the electrospinning technique include the relatively simple and inexpensive production of a large number of different types of nanofibers [20,22,50,76,77,78]. For example, by using different deposition parameters and various constructions of the spinneret, nanofibers of various morphologies, such as beads, ribbons, branched fibers, fibers with a hierarchical structure, porous, and even core-shell nanofibers can be realized (see Figure 3). For example, to form core-shell nanofibers, you can use the configuration of the spinneret, shown in Figure 4. As noted earlier, such a spinneret is made of two coaxial capillaries, through which two different, usually incompatible liquids are ejected simultaneously, forming a continuous coaxial jet.

## 3. Metal Oxide-Based Nanofibers Prepared by Electrospinning

### 3.1. General Consideration

The first attempts to form metal oxide nanofibers by electrospinning showed that it is possible to synthesize metal oxide nanofibers directly in the electrospinning process. This technology is a combination of electrospinning and the sol-gel process. However, it turned out that this process is difficult to implement due to the strict requirements of the viscoelasticity of the solution. This required careful selection of both metallic precursors and solvents and processing parameters [79,80,81]. As a result, this method was able to form only a few types of inorganic fibers, such as TiO_2_/SiO_2_ and Al_2_O_3_ [82], SiO_2_ [79], V_2_O_5_/SiO_2_ [83], SiO_2_/ZrO_2_ [84], Co_3_O_4,_ and NiO [85]. The key point of this method was to control the hydrolysis rate of sol-gel precursors by adjusting the pH value or aging conditions. However, the fibers prepared via direct electrospinning of inorganic sols were usually several hundred nanometers in diameter with poor monodispersity [86]. In addition, the ability to control the size and uniformity of the fibers was very limited due to the difficulty of accurately controlling the rheological properties of the sol [85].

In order to reduce the diameter of electrospun fibers and expand the amount of inorganic materials on the basis of which nanofibers can be formed, Li and Xia [87] suggested introducing a polymer into the sol-gel precursor. Thus, they were able to control both the viscoelastic behavior of the electrospinning solution and the sol-gel reaction. The electrospinnability of such a solution is mainly determined by the sol−gel precursor and the nature of the carrier polymer, as well as the viscosity and electrical conductivity of the solution. The carrier polymer should be spinnable, with either a high M_w_ or a significant degree of chain entanglement [88]. For the successful formation of nanofibers, the sol-gel reaction must undergo mainly in the spinning jet rather than in the stock solution [89,90]. In this case, after the solution containing polymer and metal oxide precursor is electrospun into a thin jet, the metal alkoxide immediately begins hydrolysis, reacting with moisture in the air to form a continuous gel network within the polymer matrix. This reaction produces hydroxides-polymer composite-based nanofibers. The rates of sol−gel reactions in the jet are controlled by the type of precursor used [87]. Rapid hydrolysis often causes the blockage to the spinneret, whereas rapid gelation results in a less stretchable jet and thus thicker fibers. Typically, an atmosphere with lower relative humidity and/or saturated with the solvent vapor can substantially reduce the rates of hydrolysis and gelation and therefore give rise to continuous electrospinning [85]. By optimizing these parameters, the diameter of the as-spun composite fibers can be reduced.

### 3.2. Metal Oxide Nanofibers

**Figure 6:**
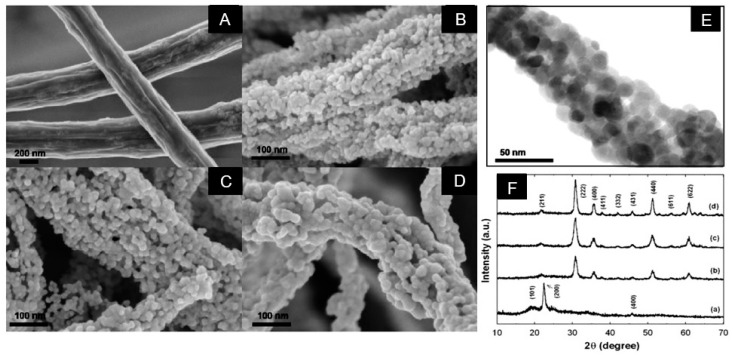
SEM images of (**A**) as prepared PVA/indium acetate composite nanofibers, (**B**) after annealing at *T*_an_ = 400 °C, (**C**) *T*_an_ = 500 °C, and (**D**) *T*_an_ = 600 °C; (**E**) TEM images of In_2_O_3_ NF after calcination at *T* = 500 °C; (**F**) XRD patterns for (a) as prepared PVA/indium acetate composite nanofibers, (b) after annealing at *T* = 400 °C, (c) *T* = 500 °C, and (d) *T* = 600 °C. Reprinted with permission from [91]. Copyright 2010 Elsevier.

**Figure 7:**
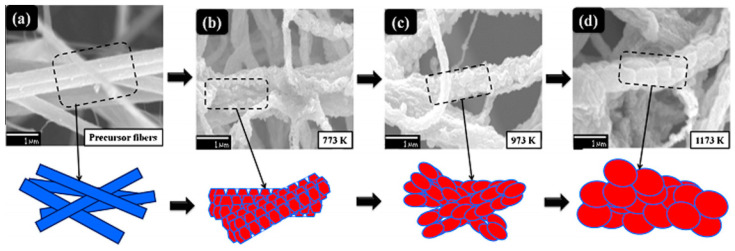
(**a**) SEM image of the composite precursor fiber (poly(styrene-co-acrylonitrile)-Zinc acetate dihydrate) and (**b**–**d**) schematic illustrations of the grain-growth mechanisms in ZnO nanofibers after calcination at *T* = 500, 700, and 900 °C. Reprinted with permission from [97]. Copyright 2014 Elsevier.

The composite nanofibers prepared by electrospinning can subsequently be converted into metal oxide nanofibers without changing their morphology via sintering at elevated temperatures. As a rule, such treatment is carried out at temperatures 500–900 °C [91]. This thermal treatment, in addition to converting hydroxides to oxides, is required to decompose and remove polymer components used for electrospinning. The transformations that occur in nanofibers after annealing are clearly visible in Figure 6. It is seen that after calcination, drastic changes in fiber morphology take place. First, after annealing, the diameter of the nanofibers decreases due to the evaporation of the polymer and solvent. Secondly, crystallization of metal oxides occurs, and the nanofiber becomes polycrystalline. The grain size in such nanofibers may vary in the range of 10–80 nm. As for the In_2_O_3_ nanofibers shown in Figure 6, then transmission electron microscopy (TEM) image presented in Figure 6 shows that the In_2_O_3_ nanofibers calcined at T = 500 °C consist of nanoparticles with a primary particle size of 10–20 nm and mesopores with a pore diameter of 10–20 nm (see Figure 6E). It is important to note that the size of the grains and crystallites in metal oxides NFs can be varied by controlling the calcination conditions (heating temperature, time, and rate). This process is well illustrated for ZnO nanofibers in Figure 7. It should be noted that the shrinkage of ceramic nanofibers due to the loss of the carrier polymer and compaction of nanocrystallites during heat treatment can lead to the rupture of ceramic nanofibers into small pieces. As a result, only small fragments of the fibers are formed, not a mat from the fibers.

As in conventional technology [92], the crystallite size in nanofibers increases with an increase in the annealing temperature [74,93,94] and annealing time [94]. For example, Wang et al. [74], during the formation of In_2_O_3_ NFs, observed an increase in the crystallite size from 10 to 23 nm with an increase in the temperature calcination from 400 to 800 °C. Dai et al. [94] reported that the size of CeO_2_ crystallites during calcination in the range of 350–900 °C increased from 10 to 36 nm. Viter et al. [95] have found that the size of SnO_2_ crystallites in electrospun fiber increased from 18.5 to 31.6 nm with an increase in the annealing time from 6 to 24 h at 600 °C. Katoch et al. [96] reported that the size of crystallites in SnO_2_–CuO composite nanofiber during annealing at 600 °C increased from 11 to 29 nm when the annealing time varied from 0.5 to 48 h.

The concentration of the metal precursor in solution, the nanofiber diameter, and the interaction of the precursor solution with the environment during electrospinning and subsequent calcination also play an important role in this process [98]. The data shown in Table 3 illustrate this effect in relation to electrospun SnO_2_ nanofibers prepared from a solution containing tin chloride pentahydrate (SnCl_4_·5H_2_O), polyvinylpyrrolidone (PVP), dimethylformamide (DMF), and ethanol [99]. The concentrations of the tin precursor in the solution were 5.5, 7, 8.5, 10, and 11.5 mM and were labeled as C0, C1, C2, C3, and C4, respectively. Solid nanofibers were then annealed at temperature 600 °C for 3 h at a heating rate of 0.5 °C/min. It is seen that the concentration really has a significant impact on all parameters of nanofibers.

As is known, for gas-sensing applications, the size of the grains and crystallites that form the NFs should be minimized. Generally, NFs with smaller grains and crystallites have better sensitivity due to the higher surface area (see Table 3). Therefore, when choosing the modes of nanofiber formation and subsequent calcination, you should take into account such a feature of nanofibers intended for use in gas sensors. At the same time, it is necessary to take into account the factor that at a too low temperature of calcination, problems arise with the complete removal of the polymer from the fiber.

At present using mentioned above electrospinning technology, nanofibers of most metal oxides used in the development of gas sensors were subsequently synthesized [100]. For example, there are reports related to nanofibers of TiO_2_ [74,101], SnO_2_ [102,103,104], WO_3_ [105], ZnO [106,107], SrTi_0.8_Fe_0.2_O_3-δ_ [108], BaTiO_3_ [21], In_2_O_3_ [91], CeO_2_ [94], CuO [93], NiO [109], etc. Table 4 gives several examples of the solvent, precursor, applied voltage and diameter of inorganic fibers formed from electrospinning.

As can be seen from Table 4, poly(vinyl pyrolidone) (PVP) and poly(vinyl alcohol) (PVA) are the most popular polymers in the fabrication of metal oxide nanofibers due to their high solubility in water and ethanol and their suitable compatibility with many salts. As a volatile solvent, one can use such solvents as ethanol, water, isopropanol, chloroform, and dimethylformamide (DMF) [45,109,110,111,112]. The choice of solution composition is based on the compatibility and solubility of a certain metal oxide precursor with a polymer solvent and the ability to achieve the required viscosity of the solution. Sometimes, in order to make the inorganic nanoparticles effectively disperse in polymer, a surfactant is needed.

### 3.3. Multicomponent Nanofibers

It was found that nanofiber composites and multicomponent nanofibers could also be formed via the electrospinning technique [14,17,23,76]. There is only one restriction. The second component needs to be soluble or well dispersed in the initial solution. If two solutions are used to form composite nanofibers, then it is necessary that each solution have the same viscosity for uniform distribution in the final product [113]. The advantage of easily forming composite nanomaterials by electrospinning gives the materials multifunctional properties optimal for a variety of applications, including gas sensor application [22,77,114]. The experiment has shown that the use of composites and multicomponent metal oxides is one of the most effective methods for improving the parameters of gas sensors [115,116]. Examples of composites and multicomponent nanofibers formed using the electrospinning technique are listed in Table 5.

### 3.4. Hollow and Core-Shell Nanofibers

The experiment showed that nanofibers with hollow and core-shell structures could also be prepared by single-nozzle electrospinning, followed by appropriate post-treatment. The ability to form hollow and core-shell nanostructures is an important factor for gas sensor application of this technology since the use of hollow spheres and nanotubes makes it possible to significantly increase the active surface of gas-sensitive materials [117].

Currently, several different mechanisms have been proposed to explain the formation of hollow fibers [118,119,120]. However, most of them recognize that for the formation of tubular structures, it is necessary that a rigid “skin” be formed before the complete removal of polymer. In this method, the morphology of the final product strongly depends on the concentration of precursor, the ratio of precursor to polymer, the calcination temperature, and heating rates. Nanotubes of gas-sensitive metal oxides such as CeO_2_ [94], Y_2_O_3_-ZrO_2_ [121], ZnO [122], TiO_2_ [123], BaFe_12_O_19_ [124], α-Fe_3_O_4_ and Co_3_O_4_ [125], Fe_2_O_3_ [119], CoFe_2_O_4_ [126], CuO [127], and SnO_2_ [120] have been prepared by this method.

**Figure 8:**
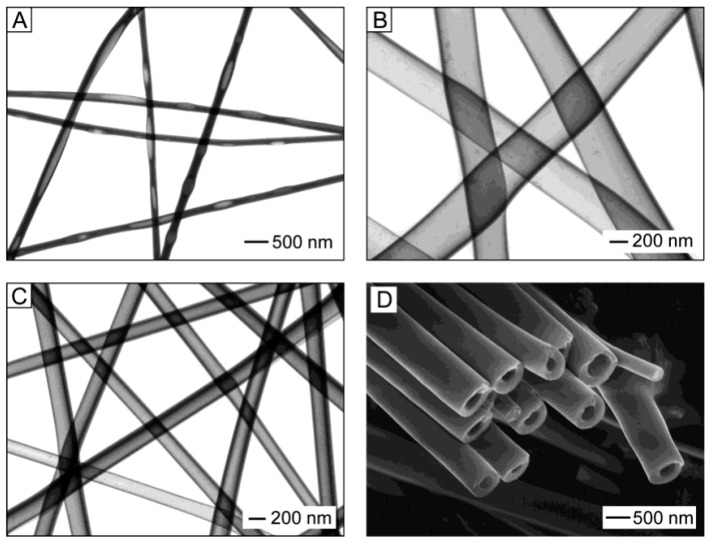
(**A**–**C**) TEM images of TiO_2_/PVP hollow fibers fabricated by electrospinning with various injection rates for the oil phase and different voltages: (**A**) 0.03 and (**B**) 0.3 mL/h under a voltage of 12 kV; and (**C**) 0.1 mL/h and 16 kV. The sheath liquid was an ethanol solution that contained both Ti(OiPr)_4_ (0.3 g/mL) and PVP (0.03 g/mL). (**D**) SEM image of TiO_2_/PVP hollow fibers prepared by electrospinning a PVP solution (in ethanol, 0.03 g/mL) that contained 0.5 g/mL of Ti(OiPr)_4_. Reprinted with permission from [128]. Copyright 2004 ACS.

Experiments carried out by Li and Xia [55,128] have shown that coaxial electrospinning of two immiscible liquids through a coaxial, two-capillary spinneret is the most suitable technology for forming hollow fibers (see Figure 4). Li and Xia [55,128] offered to fabricate TiO_2_ hollow fibers by co-electrospinning viscous heavy mineral oil as the core and a mixture ethanol solution of PVP and Ti(OiPr)_4_ as the shell. They found that rapid stretching of the sheath causes strong viscous stress that stretches the oil phase and lengthens it along with the sheath solution through viscous entrainment and/or contact friction mechanisms. As a result, the heavy mineral oil remains in the shell of the amorphous TiO_2_/PVP composite. That is why the removal of the mineral oil contributes to the subsequent formation of TiO_2_/PVP composite tubes, which, after calcination at elevated temperatures (T = 500 °C) in the air, transform into hollow TiO_2_ fibers. The oil can be extracted by immersing the sample in octane. The wall thickness and inner diameter of the hollow nanofibers could be varied in the range from tens of nanometers to several hundred nanometers by controlling the processing parameters such as the applied voltage and the injection rate for the oil phase (see Figure 8).

Li and Xia [55,128] showed that the wall thickness of the resulting hollow TiO_2_ fibers could also be controlled by varying the concentration of the alkoxide introduced into the PVP solution. It is important to note that the attempts to replace the mineral oil with some polymer solutions while using the same solution for the sheath were unsuccessful. In this case, no hollow structure was observed, although the core liquid was sufficiently viscous to be electrospun as nanofibers. This result implies that the core and sheath solutions were completely mixed during the electrospinning process. Hollow fibers of metal oxides such as TiO_2_, WO_3_, In_2_O_3_, LiNiO_2_, LiCoO_2_, BaTiO_3,_ and SnO_2_ were prepared by using mentioned above approach [9,86,129,130,131,132,133].

Using the coaxial co-electrospinning technique shown in Figure 4, core-shell structures can also be formed. To form the core region, instead of mineral oil, a composite solution is used, which allows the deposition of metal oxide nanofibers. If the precursors intended for the formation of core and shell regions allow the formation of various metal oxides, then, in the end, we obtain a core-shell structure. Koo et al. [134], using this approach, prepared In_2_O_3_/α-Fe_2_O_3_ core/shell nanofibers. To fabricate the core region, indium (III) chloride tetrahydrate, dissolved in ethanol and PVP dissolved in a DMF:ethanol solution was used, while for the shell region, the solution of iron (III) nitrate nonahydrate dissolved in DMF and PVP was used. For metal oxide crystallization and removal of residual polymer, as-electrospun NFs were heat-treated at 500 °C for 5 h.

**Figure 9:**
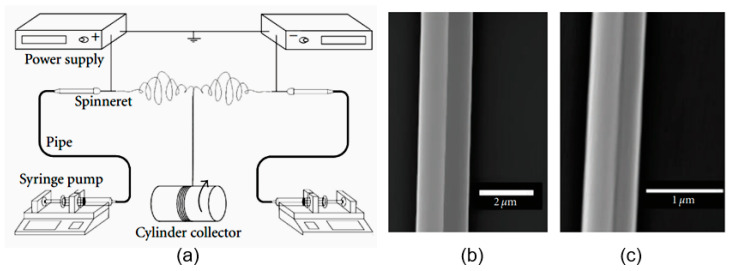
(**a**) Schematic diagram of dual-opposite-spinneret electrospinning apparatus. (**b**,**c**) Detailed microstructure of side-by-side fiber by FE-SEM (**b**) as-electrospun composite fiber and (**c**) calcined TiO_2_–SnO_2_ nanofiber. Reprinted from [135].

Xu et al. [135] demonstrated another interesting approach to the formation of metal oxide nanofibers. Using dual-opposite-spinneret electrospinning (see Figure 9a), they produced well-aligned and uniform side-by-side TiO_2_–SnO_2_ fibers (see Figure 9b). Two spinnerets were assembled horizontally in opposite directions, and each was connected to a separate high-voltage power supply. A rotating cylinder covered with aluminum foil was used as a collector. The distance between the tips of two spinnerets was 12 cm, the applied voltages were +3100 V and −3100 V, the distance between the spinnerets and the collector was about 15 cm, the rotation rate of the cylinder collector (Diameter 10 cm) was 300 r/min. Solutions for electrospinning were prepared by dissolving polyvinylpyrrolidone (PVP) and tetrabutyl titanate or stannous octoate into a mixed solvent of ethanol and acetic acid (4:1). After electrospinning, the electrospun fibers were calcined at 500 °C for 2 h in air. The diameter of electrospun fibers before calcining was ~1.75 μm. After calcining, the diameter of nanofibers became much smaller, only ~0.96 μm. Such structures have not been used in the manufacture of gas sensors. However, it is quite possible that such an unusual configuration of metal oxides in a gas-sensitive matrix can provide unexpected results.

### 3.5. Porous Nanofibers

When developing gas-sensitive materials, one of the most important tasks is to increase their porosity, i.e., improving their gas permeability. The structure of the nanofibers array itself provides high gas permeability of the nanofiber-based mat forming the gas-sensitive layer. However, this may not apply directly to nanofibers, which, under certain conditions of synthesis, can have a sufficiently dense structure that prevents rapid diffusion of gas into the fiber. Therefore, the ability to manufacture porous nanofibers during their formation is an important advantage of electrospinning technology [75]. The presence of pores also contributes to an increase in the active surface area, which is extremely important for achieving the high sensitivity of gas sensors [12].

Currently, two methods are mainly used to obtain the porous structure of nanofibers. One of them controls the electrospinning environment and the interaction of the solution and influences the fiber structure through the “breath figure” (BF) [136,137] and “phase separation” mechanisms [69,138], while the other uses a sacrificial material as a pore generator. The “breath figure” process is controlled by complex heat and mass transfer, and these transfer processes are additionally dependent on various experimental parameters such as temperature, humidity, air velocity, physical properties of solvents and solution, and physical and chemical properties of polymers. A slight change in any of these parameters can significantly change the size and shape of the pores.

Phase separation during electrospinning can be applied to introduce a porous structure by transforming one phase as the pores while the other as the nanofiber matrix. In this case, phase separation can occur (i) between the polymer and solvent or (ii) between the polymer and a nonsolvent [17]. Phase separation is a complex phenomenon that depends on the molecular parameters of the spinnable solution, such as the miscibility of the two polymers, their concentration, and the solvent used [138]. Thermodynamic parameters such as composition, temperature, pressure, and other processing parameters [138] also have a significant effect on the pore formation process. For example, phase separation between the polymer and solvent can be induced by rapidly cooling the incompletely solidified jet.

Some aspects of the formation of porous nanofibers, such as the effect of temperature, air humidity, and solvent volatility, were discussed earlier in Section 2.2, and some will be discussed in Section 4.2 and in the second part of this article. More details on the specifics of pore formation in nanofibers formed by electrospinning can be found in [17,75]. It is important to note that these pore formation methods were developed for polymer nanofibers [68,73,139,140,141]. However, they can be successfully applied in the formation of porous metal oxide electrospun fibers using a polymer-sol-gel solution since the highly porous structure is preserved after calcination.

## 4. Approaches to Optimization of Nanofibers’ Parameters

The experience gained in the development of conventional gas sensors has shown that pristine metal oxides cannot always meet all the requirements arising in the development of sensors designed for a specific application. As a rule, to solve this problem, these metal oxides have to be modified to provide them the desired properties [142,143,144,145]. For example, to increase the catalytic activity of metal oxides, their surface is modified with clusters of noble metals [146,147]. Research has shown that the same problems arise in the development of nanofiber-based gas sensors. The experiment showed that for the functionalization of nanofibers, all methods developed for metal oxides could be used, including decoration with clusters of noble metals. If we systematize them, then all methods used to optimize the parameters of nanofibers can be divided into blending and post-modification methods.

### 4.1. Blending

**Figure 10:**
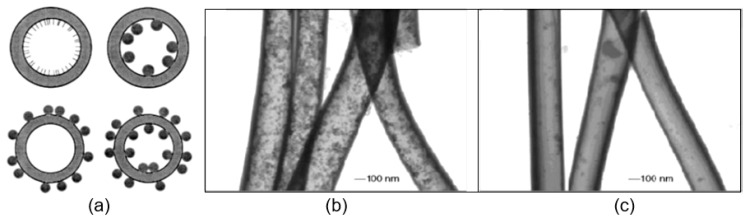
(**a**) Schematic drawings showing cross-sections of hollow nanofibers whose surfaces are derivatized with functional molecules (the top plate) and nanoparticles (NPs) (the other plates). (**b**,**c**) TEM images of hollow titania nanofibers immersed in an oil-based ferrofluid overnight. The hollow fibers are prepared by co-electrospinning, either with (**b**) or without (**c**) octadecyltrichlorosilane (OTS) added to the mineral oil. The interior of a hollow fiber is decorated by oil dispersible Au nanoparticles. Reprinted with permission from [149]. Copyright 2005 Wiley.

The blending method refers to the process that is to modify the properties of nanofibers by adding modifying additives directly to the electrospinning solution. For example, for the modification of metal oxides with noble metals, noble metal NPs or their precursors are mixed with the spinning solutions to form a uniform precursor solution, and then the mixed solution is directly electrospun to form after calcination various materials interfaces. This method has advantages such as easy preparation and high yield, and it is widely used to improve the sensitivity and selectivity of gas-sensitive materials [148]. However, it should be borne in mind that direct dispersing the functional agents into the polymer solution for electrospinning may be accompanied by its aggregation. Therefore, additional treatments such as ultrasonic dispersion of a solution or manifold repetition of blending are required when preparing a solution for electrospinning. Suitable results are also obtained by introducing modifying agents with a surface-active agent into the solution, which prevents the aggregation of nanoparticles. If we use coaxial electrospinning to make, for example, hollow nanofibers, then by adding functional particles into the core liquid, which will be removed later, hollow nanofibers with decorated surfaces can be obtained [129,149]. The resulting hollow fibers are shown in Figure 10. Li et al. [149] showed that many types of surfactant-protected nanoparticles could form stable dispersions in mineral oil.

The blending method, in addition to decoration, also allows the doping of metal oxides. For example, using this approach, Lin et al. [150] fabricated aluminum-doped zinc oxide (AZO) nanofibers. The concentration of the doping element Al in ZnO was controlled by adding aluminum nitrate to solutions for electrospinning. The addition of Al (0–3.0 at.%) made it possible to reduce the diameter of AZO nanofibers and make them more uniform, which is very important for various applications, including gas sensors. Zhang et al. [151], in the same way, through doping with magnesium (1–6 mol%), influenced the electrical conductivity of In_2_O_3_ nanofibers. Mg-doped In_2_O_3_ NFs were prepared using InCl_3_·4H_2_O and Mg(NO_3_)_2_·6H_2_O precursors mixed with PVP and DMF. Calcination was performed at 600 °C. Other doping additives used in the optimization of gas sensor parameters are listed in Table 6. The concentration of doping additives in the formed metal oxide nanofibers ranged from 0.05 to 10 wt.%. However, as a rule, the maximum sensor effect, as will be shown later, was observed at a concentration of 1–5 wt.%, and in some cases, at an even lower concentration of dopants.

### 4.2. Post-Modification

In post-modification, metal oxide nanofibers are prepared by electrospinning, and only after that are they subjected to processing, which affects the properties of the formed nanofibers. For example, to decorate the surface of metal oxides with clusters of noble metals, metal NPs can be adsorbed from solutions or deposited by various physical and chemical methods onto metal oxide nanofibers to obtain the metal NP-NF-based interface. In particular, for these purposes, you can use the physical dip-coating method, which is one of the simplest methods to endow nanofibers with active sites for target interaction. This process can also include post-treatment, such as calcining. For example, Wang et al. [152], to obtain Pd^0^–SnO_2_ composite-based nanofibers, proposed the process with the following stages: initially, Pd–SnO_2_ composite was formed by the blending method using SnCl_2_ and PdCl_2_ precursors through the electrospinning. After that, the composite fibers were heat-treated to obtain Pd^2+^-loaded SnO_2_ composite fibers, which were finally transformed in Pd^0^–SnO_2_ under the reduction by hydrazine hydrate.

The principles of post-modification of nanofibers can also be used to form hollow and core-shell metal oxide structures. To date, several approaches have been developed for the manufacture of hollow metal oxide nanofibers using the principles of post-treatment. For example, electrospun nanofibers can be used as sacrificial templates for making tubular fibers [153,154]. In this case, as fiber templates, one can use either polymer [155] or carbon nanofibers (CNTs) [156]. Metal oxide tubes can be obtained after coating and removing the template fibers.

**Figure 11:**
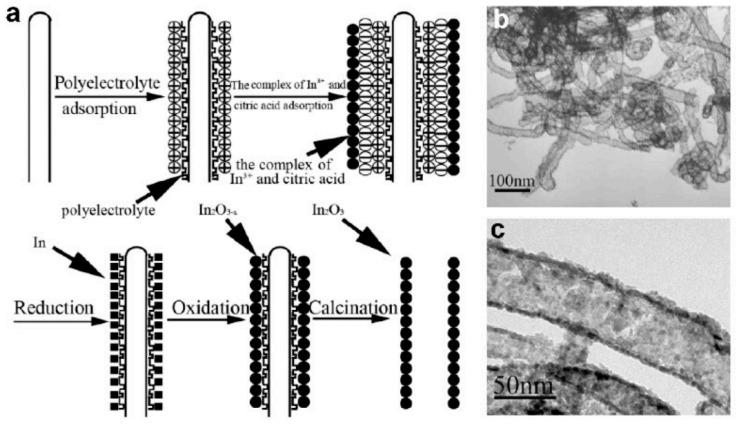
(**a**) Schematic diagram for the growth process of In_2_O_3_ hollow nanofibers. (**b**,**c**) TEM images of regular In_2_O_3_ nanotubes prepared by the calcination of In_2_O_3_/polyelectrolyte/CNT nanocomposites at 550 °C in O_2_ for 3 h. Reprinted with permission from [156]. Copyright 2007 Wiley.

Various coating techniques, such as chemical and physical vapor deposition, sol-gel process, layer-by-layer assembly, electrochemical deposition, etc. [153,154,199,200], can be used for preparing hollow and core-shell metal oxide structures. The templates can be removed by heat treatment [153,199] or solvent extraction [154] to obtain tubular structures. For example, Du et al. [156] fabricated porous In_2_O_3_ hollow nanofibers using a layer-by-layer assembly technique and CNTs as a template. A diagram of this process is shown in Figure 11. After several cycles of reduction and oxidation reactions carried out on the surface of CNTs, and the subsequent removal of the CNT template by annealing in O_2_ at 550 °C during 3 h, hollow In_2_O_3_ nanofibers were fabricated. These In_2_O_3_ hollow nanofibers had a diameter from 30 to 60 nm [156]. It is important that the walls of nanofibers had nanopores, which were formed during the removal of the CNTs by the calcination process. These nanometer-sized pores allow the test gas to penetrate deep into the gas-sensitive material, which significantly increases the surface-to-volume ratio of the fabricated In_2_O_3_ hollow nanofibers. This property of the nanofibers formed was of great importance for the gas-sensing effect [156]. Due to increased surface area and open porous structure, sensors had increased sensitivity and a better rate of response.

**Figure 12:**
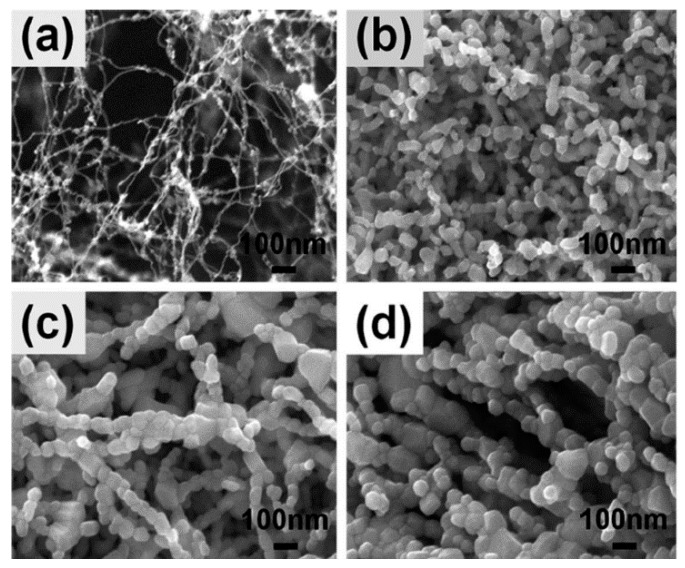
(**a**) The morphology of the SWCNT template. (**b**–**d**) WO_3_ nanowire morphology for W deposition times of 10 s, 20 s, and 60 s, respectively. The WO_3_ structures were fabricated by oxidation at 700 °C in air for 2 h. Reprinted with permission from [201]. Copyright 2012 Royal Society of Chemistry.

It should be borne in mind that when using physical methods of coating template fibers, as a rule, it is not possible to form hollow nanofibers. These methods cannot provide the coating of all sides of the template. Therefore, after removing the template, we obtain ordinary nanofibers, the morphology of which depends on the morphology of the original template and the coating modes. Figure 12 shows WO_3_ nanofibers prepared by deposition of W on SWCNT template with the following annealing in an oxygen-containing atmosphere [201].

Fan et al. [202] showed that the surface area of hollow NFs could be even larger if NFs are formed with a branch-on-stem morphology. To do this, they developed a fabrication strategy that included electrospinning of PVP NFs followed by etching of these fibers with oxygen plasma. The result was a hierarchical template that could be used to deposit SnO_2_ film. The surface morphology of the template depended on sputtering time. After removing the template, the SnO_2_ NFs had uniformly distributed branches all over the stem.

**Figure 13:**
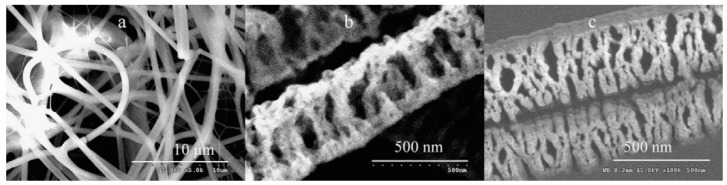
SEM images of polymer fiber after different post-treatment: (**a**) without post-treatment; (**b**) oxygen plasma etching; and (**c**) oxygen plasma etching and 500 °C annealing for 4 h. Reprinted with permission from [203]. Copyright 2010 Elsevier.

Previously, Zhang et al. [203] also used oxygen plasma treatment of nanofibers, but the purpose of this treatment was to increase the porosity of metal oxide nanofibers formed directly during electrospinning. As is known, the high porosity of a gas-sensitive material is one of the conditions for achieving high sensitivity of sensors with suitable response time. Using the SnO_2_ nanofibers as an example, Zhang et al. [203] demonstrated that oxygen plasma etching of as-electrospun poly(vinyl alcohol) (PVA)/SnCl_4_·5H_2_O composite fibers before calcination gives a significant increase in their porosity, which is retained even after annealing (see Figure 13).

It is seen that, after annealing at 500 °C, no obvious changes in the fiber morphology occur. The plasma reaction system worked at a frequency of 13.56 MHz. Zhang et al. [203] found that the etching power of 50 W and the etching time of 30 min are the optimal etching conditions. With an increase in the etching time to 90 min, the porous microstructure of the fibers was destroyed. By increasing the porosity, it was possible to achieve a higher sensitivity of SnO_2_ sensors to ethanol. At 340 °C, the detection limit to ethanol was <1 ppb. Most likely, the high sensitivity is a consequence of the small size of the SnO_2_ crystallites in the nanofiber, which did not exceed 7 nm. The highly porous structure of nanofibers, due to a decrease in the number of contacting crystallites, hindered the growth of crystallites during annealing. Oxygen plasma etching also increased sensor response of CuO/SnO_2_ sensors to H_2_S [204].

To increase the porosity of metal oxide nanofibers, one can use other methods developed for polymer nanofibers [17]. For example, you can use methods based on electrospinning mixtures with subsequent selective removal of one phase or on phase separation based on solvent evaporation [55,205]. Porous fibers can also be obtained using a highly volatile solvent [206].

As for the formation of core-shell metal oxide structures using post-treatment principles, the approaches that allow the creation of such structures does not differ in any way from the approaches described earlier when discussing the processes of formation of hollow nanofibers. The only difference is that instead of polymer or carbon nanofibers, metal oxide nanofibers formed by electrospinning are used as templates, and various chemical methods of forming metal or metal oxide coatings are used as post-treatment. If metals are deposited on the surface of metal oxide nanofibers, then subsequently, the structures undergo thermal oxidation. The most common methods used in the formation of core-shell structures are electrochemical deposition and hydrothermal, sol-gel, sonochemical, and microwave assistance synthesis [207].

## 5. Summary

The analysis showed that electrospinning technology really presents great opportunities for the formation of gas-sensitive materials with a unique combination of parameters. Electrospinning makes it possible to form a gas-sensitive matrix from small crystallites while maintaining a very high gas permeability of the matrix due to the ultra-high porosity of the structure. This, on the one hand, should provide ultra-high sensitivity of the sensors under optimal conditions, and on the other hand, guarantee a fast response and recovery since such a structure has no diffusion restrictions for gas penetration into the gas-sensitive matrix. This technology is also effective for the manufacture of hollow tubes and core-shell structures, the use of which provides additional opportunities for improving the parameters of conductometric gas sensors. The nanofiber-based conductometric gas sensors are discussed in more detail in the second part of the article.

## Figures and Tables

**Table 1:** Polymers and solvents commonly used for electrospinning.

Polymer	Solvent	Weight of Polymer, wt%
PEO	Water	1–4
	DMF	7
	Chitosan/water (1:1)	2
PVA	Water	12
	Ethanol/water (1:1)	8–10
PVP	Ethanol	4.50
	Water	10
	DMF	14
PMMA	Tetrahydrofuran	10
	Acetone	10
	CHCl_3_	10
PS	CHCl_3_	20
	Tetrahydrofuran	20

DMF—dimethylformamide; PEO—poly(ethylene oxide); PMMA—polymethyl methacrylate; PS—polystyrene; PVA—poly(vinyl alcohol), PVP—polyvinylpyrrolidone; sources: data extracted from [48].

**Table 2:** Electrospinning parameters (solution, processing, and environment) and their effect on fiber morphology.

Parameters			Effect on Fiber Morphology
Solution parameters	Viscosity	Low	Discontinuation of filament formation, beads formation
		High	Increase in fiber diameter, disappearance of beads, spinning prevention
	Polymer concentration		Increase in fiber diameter with increase in concentration
	Molecular weight of polymer	Lower	Larger deposition area, smaller fibers, bead formation, appearance of droplets
		Higher	Smaller deposition area, larger fibers
	Conductivity		Decrease in fiber diameter with increase in conductivity
	Surface tension		No conclusive link with fiber morphology, high surface tension results in instability of jets
Processing parameters	Applied voltage		Decrease in fiber diameter with increase in voltage
	Distance between tip and collector		Generation of beads with too small and too large distance, optimal distance is required for uniform fibers
	Flow rate	Low	Decrease in fiber diameter
		High	Generation of beads with too-high flow rate
Ambient parameters	Humidity	Low	Broken filaments, nozzle clogging
		High	High humidity results in circular pores on the fibers
	Temperature	Low	High viscosity and larger fiber diameter, nozzle clogging
		High	Less viscosity and smaller fiber diameter, uniform formation of fibers

**Table 3:** Properties of the solution for electrospinning and the resulting nanostructures after annealing.

Sample Label	Precursor Concentration, mM	Solution Viscosity, cP	Surface Area, m^2^/g	Diameter, nm	Crystallite Size, nm
C0	5.5	256.7	7	114–170	18–27
C1	7.0	298.1	78	124–191	5–15
C2	8.5	304.9	12	162–224	15–25
C3	10	321.5	10	170–195	20–40
C4	11.5	344.2	10	213–337	30–75

Source: Reprinted with permission from [99]. Copyright 2014 RSC.

**Table 4:** Examples of solvents and precursors used to make metal oxide-based nanofibers.

Metal Oxide	Precursor	Polymer	Solvent	Diameter, nm
SnO_2_	SnCl_2_·2H_2_O	PVA	Water:1-propanol:isopropanol	80–400
	Tin (IV) acetate	PVAc	DMF	
	SnCl_2_·2H_2_O	PVP; PAN; PVB	DMF or DMF:ethanol	
	SnCl_4_·5H_2_O	PVA;	DI water	
In_2_O_3_	In(NO_3_)_3_·xH_2_O	PVP	DMF or DMF:ethanol = 1:1	40–300
	InCl3·4H_2_O		DMF	
TiO_2_	TTIP	PVP	Acetic acid:ethanol	30–500
	TTB		DMF:ethanol	
ZrO_2_	Zr(AC)	PVAc	DMF	200
CeO_2_	(NH_4_)_2_Ce(NO_3_)_6_	PVP	Water:ethanol	50–1000
	Ce(acac)_3_		Acetone	
	Ce(NO_3_)_3_	PVA	Water:ethanol	
	Ce(CH_3_COO)_3_	PEO	Water	
ZnO	Zn(AC)_2_·H_2_O	PVP	Ethanol	50–250
		PS-co-acrylics	DMSO	
		PVP	DMF	
WO_3_	WCl_6_	PVP	DMF:ethanol	20–250
	(NH_4_)_6_[H_2_W_12_O_40_]nH_2_O		Water	
	H_2_WO_4_ + H_2_O_2_		Ethanol	
Co_3_O_4_	Co(NO_3_)_2_·6H_2_O	PVP	DMF:ethanol = 1:1	100–200
CuO	Cu(CH_3_COO)_2_	PVP	Ethanol	70–1400
	Cu(CH_3_COO)_2_	PVA	Water	

DMF—dimethylformamide; DMSO—dimethyl sulfoxide; (NH_4_)_2_Ce(NO_3_)_6_—ceric ammonium nitrate; PS-co-acrylics—poly(styrene–co-acrylonitrile); TTIP—titanium tetraisopropoxide; TTB—titanium tetrabutoxide; Zn(AC)_2_·H_2_O—Zn(CH_3_COO)_2_·2H_2_O—zinc acetate; Zr(AC)—Zr(C_8_H_12_O_8_)—zirconium acetate.

**Table 5:** Examples of solvents and precursors used to make composite- and multicomponent-based metal oxide nanofibers.

Metal Oxide	Precursor	Polymer	Solvent
In_2_O_3_–CeO_2_	In(NO)_3_ 4H_2_O and Ce(NO)_3_ 6H_2_O	PVP	DMF
Al_2_O_3_–In_2_O_3_	In(NO)_3_ 4H_2_O and Al_2_(NO)_3_	PVP	DMF
In_2_O_3_–SnO_2_	In(NO)_3_ and SnCl_2_	PVP	DMF:ethanol
In_2_O_3_/TiO_2_	C_16_H_36_O_4_Ti and In(NO)_3_ 4H_2_O	PVP	Acetic acid:ethanol
NiO/SnO_2_	Nickel chloride hexahydrate and tin chloride dehydrate	PVP	DMF:ethanol
ZnO/SnO_2_	Zinc nitrate and tin chloride dehydrate	PAN	DMF
	Zn(NO_3_)_2_·6H_2_O and SnCl_2_·2H_2_O	PVP	DMF:ethanol
	SnCl_2_·2H_2_O and ZnCl_2_		DMF:ethanol
	Zinc acetate and tetraethyl orthosilicate		DMF, DMSO ^1^, HCl and ethanol
NiTiO_3_	Nickel acetate and titanium isopropoxide	PVP	Methanol:acetic acid
MgTiO_3_	Magnesium ethoxide and titanium isopropoxide	PVAc	2-methoxyethanoland DMF
CoFe_2_O_4_	Co(NO_3_)_2_·6H_2_O and Fe(NO_3_)_3_·9H_2_O	PVAc	DMF:THF ^2^
ZnCo_2_O_4_	Zn(NO_3_)_2_ and Co(NO_3_)_2_	PVP	Ethanol

^1^ DMSO—dimethyl sulfoxide; ^2^ THF—tetrahydrofuran.

**Table 6:** Dopants used for optimization of nanofiber-based gas sensors’ response to specific analyte gas.

Material	Dopant	Analyte Gas	T_oper_., °C	Ref.
SnO_2_	Y; Ni	Acetone	300–340	[157,158,159]
ZnO	Ce; Mn; Co; La		230–360	[160,161,162,163,164]
WO_3_	La; Cu		300–350	[165,166]
In_2_O_3_	Eu		240	[167]
α-Fe_2_O_3_	La; Nd; Sm; Ce; Ca		200–240	[168,169,170,171,172]
SnO_2_	Cu; Pr; Sr; Yb; Ce; Fe; Co	Ethanol	260–300	[173,174,175,176,177,178,179]
ZnO	In; Er; Al; Cr; Ce		240–300	[162,163,180,181,182]
In_2_O_3_	Co; Mg; Eu		250–300	[183,184,185]
α-Fe_2_O_3_	Ca; Sm; Nb		200–240	[172]
SnO_2_	Al	Formaldehyde	240	[186]
In_2_O_3_	Er; Nd; Sm		240–260	[187,188,189,190]
α-Fe_2_O_3_	Sn		220	[191]
ZnO	Ni	Acetylene	250	[192]
NiO	W	Xylene	375	[193]
SnO_2_	Al; Co	Hydrogen	330–340	[194,195]
SnO_2_	Cu	Hydrogen sulfide	125	[196]
ZnO	Cu		230	[197]
In_2_O_3_	V		90–150	[198]

## Data Availability

All the data are reported in the paper directly.
